# Machine learning adaptation of intraocular lens power calculation for a patient group

**DOI:** 10.1186/s40662-021-00265-z

**Published:** 2021-11-15

**Authors:** Yosai Mori, Tomofusa Yamauchi, Shota Tokuda, Keiichiro Minami, Hitoshi Tabuchi, Kazunori Miyata

**Affiliations:** 1 Miyata Eye Hospital, 6-3 Kurahara-cho, Miyakonojo, Miyazaki 885-0051 Japan; 2 Department of Ophthalmology, Tsukazaki Hospital, 68-1 Waku, Aboshi-ku, Himeji, Hyogo 671-1227 Japan; 3 Department of Technology and Design Thinking for Medicine, Hiroshima University, 1-2-3 Kasumi, Minami-ku, Hiroshima, 734-8553 Japan

**Keywords:** Machine learning, Adaptation, Intraocular lens power calculation, Patient ethnicity, Patient race, Region of patient, SRK/T formula

## Abstract

**Background:**

To examine the effectiveness of the use of machine learning for adapting an intraocular lens (IOL) power calculation for a patient group.

**Methods:**

In this retrospective study, the clinical records of 1,611 eyes of 1,169 Japanese patients who received a single model of monofocal IOL (SN60WF, Alcon) at Miyata Eye Hospital were reviewed and analyzed. Using biometric metrics and postoperative refractions of 1211 eyes of 769 patients, constants of the SRK/T and Haigis formulas were optimized. The SRK/T formula was adapted using a support vector regressor. Prediction errors in the use of adapted formulas as well as the SRK/T, Haigis, Hill-RBF and Barrett Universal II formulas were evaluated with data from 395 eyes of 395 distinct patients. Mean prediction errors, median absolute errors, and percentages of eyes within ± 0.25 D, ± 0.50 D, and ± 1.00 D, and over + 0.50 D of errors were compared among formulas.

**Results:**

The mean prediction errors in the use of the SRT/K and adapted formulas were smaller than the use of other formulas (*P* < 0.001). In the absolute errors, the Hill-RBF and adapted methods were better than others. The performance of the Barrett Universal II was not better than the others for the patient group. There were the least eyes with hyperopic refractive errors (16.5%) in the use of the adapted formula.

**Conclusions:**

Adapting IOL power calculations using machine learning technology with data from a particular patient group was effective and promising.

## Background

Premium intraocular lenses (IOLs), such as toric and presbyopia-correcting IOLs, necessitate accurate power calculations to minimize postoperative refraction error. With the use of third- and fourth-generation calculations, such as the SRK/T and Haigis formulas, postoperative refractive errors fall within ± 1.00 D in 93% of eyes [1], which is acceptable for the use of monofocal IOLs. Higher accuracy of over 90% within ± 0.50 D error is desired for most patients to obtain uncorrected distance visual acuity of 20/20 or better. Currently, sophisticated power calculations, such as the Barrett Universal II (BUII) [2] and Hill-Radial Basis Function (Hill-RBF) [3], have been recommended, and their superiority were demonstrated in several publications [4–6]. New generation formulas enable higher accuracy by adding more biometric measurements, such as lens thickness and corneal diameter, utilizing complicated modeling of ocular geometry, and utilizing machine learning with a large dataset.

There are prediction errors inherent to a patient’s ethnicity, race, and region, since norms in Caucasian eyes are assumed in most ocular modeling, and large training data are obtained from Caucasian eyes. Fundamentally, an ocular optical system is determined by the relative position and properties of the cornea, anterior segment, crystalline lens, and ocular axial length, and thus characteristics of ocular optics varies with geometric or anatomical differences in different patient groups. Compared with Caucasian eyes, Chinese corneas show flatter keratometry and more prolateness [7], and smaller corneal diameters and shallower anterior chambers are found in Chinese and Japanese eyes [8]. Such geometric differences influence the assumption used in conventional formulas. Assessment by Melles et al. with 18,501 implanted eyes shows that the performances of power calculation formulas vary with axial length (AXL), anterior chamber depth (ACD), mean keratometry (K), and lens thickness (LT) [6]. The influences of ACD and LT on the performance of recently developed formulas are revealed in 695 Caucasian eyes [9]. In fact, there are particular characteristics in the patient group of each site. For example, cataract patients on Kyushu Island in Japan are characterized by smaller corneal diameters, shallower anterior chambers, and narrower angles [10, 11]. Hence, it is important to adapt conventional and universal power calculations for patients.

Conventionally, the differences among different patient groups have been adjusted by optimizing the constants of formulas. Constant optimizations are possible in the third- and fourth-generation formulas. However, optimizations of unpublished formulas, such as the BUII and Hill-RBF, are impractical [12]. In addition, these calculations are optimized for major IOL models. Therefore, it is a major concern that comparable accuracy might be achieved in the use of other IOL models, which are available and common for regional medical situations.

Alternatively, machine learning technology has been utilized to construct power calculators that are adapted for a patient group. In the field of IOL power calculations, several machine-learning based calculations have been developed, such as the Hill-RBF [3], Kane [13], Sramka [14], and Pearl-DGS [15]. Support vector regression (SVR) is a machine learning technique that provides a nonlinear regression function that have at most a certain margin from actually obtained targets (corresponding to prediction errors) for all training data and was as flat as possible (corresponding to minimum amounts of regression coefficients) [16]. Carmona González et al. combined SVR and multivariate adaptive regression spline with training set data using 208 eyes [17], providing the most accurate performance compared with the 4 conventional formulas. Ladas et al. revealed that SVR supervised nonlinear regression machine learning was suitable for optimization of existing IOL power calculation formulas, compared with extreme gradient boosting (XGBoost) and artificial neural network (ANN) [18]. In the evaluation of various machine learning algorithms with a training dataset of 2,831 eyes of 1,659 patients with 13 kinds of IOLs by Yamauchi et al., superior performances were obtained with SVR over conventional formulas [19]. While the sample size was insufficient and/or multiple IOL models were used for training and evaluation, the previous approaches demonstrated the potential of machine learning for obtaining a power calculation suitable for a particular patient group. This retrospective study aimed to examine the effectiveness of machine learning-based power calculations for a patient group using a single IOL model.

## Methods

### Participants

This study was approved by the institutional review board of Miyata Eye Hospital (CS-334) and adhered to the tenets of the Declaration of Helsinki. The use of clinical records related to cataract surgery were approved via informed consent before surgery. Clinical records of 1792 eyes of 1269 consecutive Japanese patients who underwent cataract surgery at Miyata Eye Hospital with implantation of the IOL SN60WF (Alcon, Fort Worth, TX) from November 2017 until July 2019 were reviewed. The inclusion criteria were eyes in which AXL, K with diameters of 2.5 mm, ACD, LT, central corneal thickness (CCT), and white-to-white width (WTW) were measured preoperatively using an OA-2000 swept-source biometer (Tomey, Nagoya, Japan). Eyes in which the postoperative corrected distance visual acuity was worse than 16/20 were excluded from analysis.

**Fig. 1:**
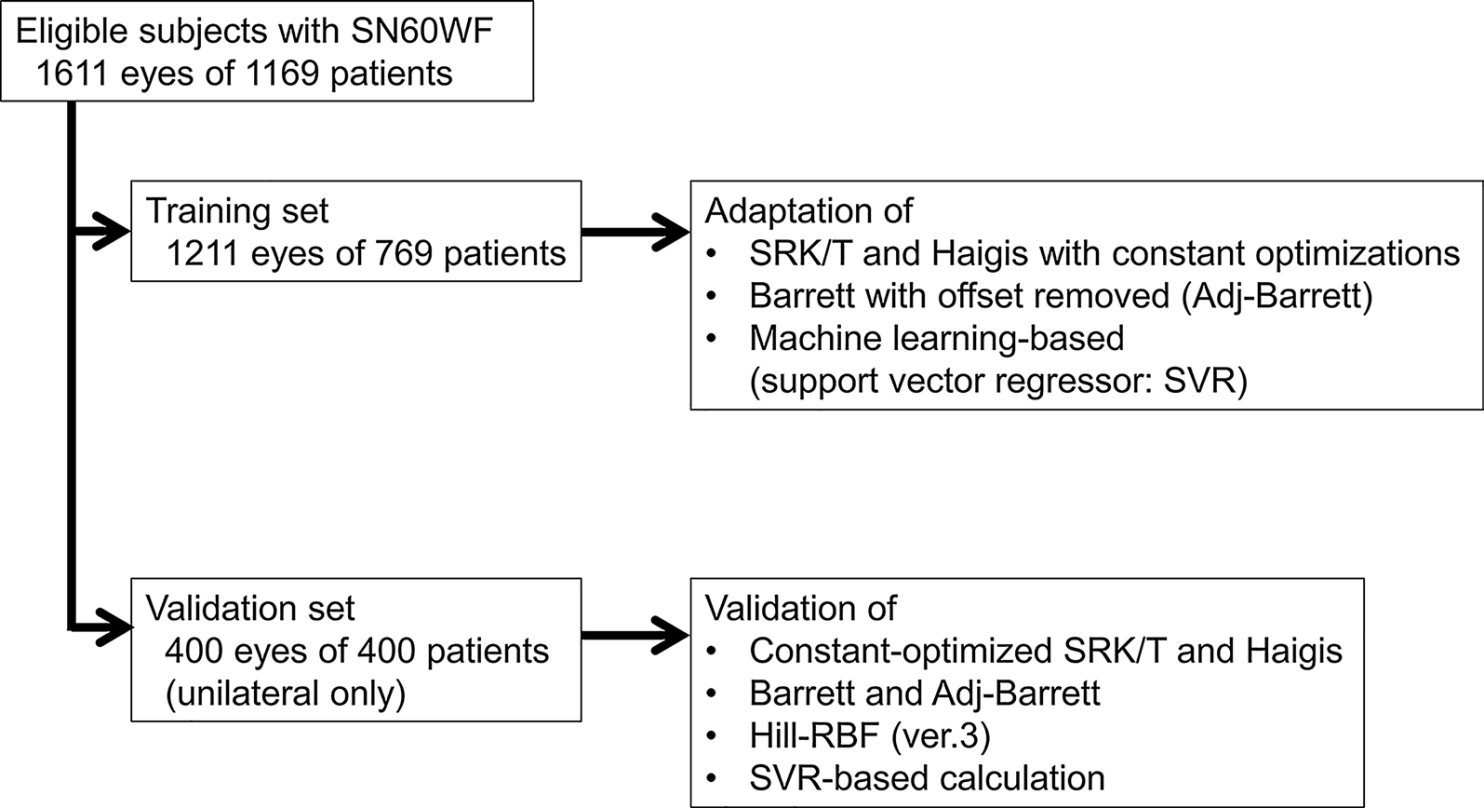
Training and validation sets assigned from eligible subjects for adapting and evaluating the performance of power calculation formulas for a patient group

Subjects were divided into two groups (Fig. 1). A training set was used for adapting power calculations for the patient group. A validation set was used to evaluate the effect of the adaptations. Manifest refraction spherical equivalent (MRSE) at 3 months postoperatively were obtained during the best-corrected visual acuity examination at 5 m by experienced examiners. Prediction errors of predicted postoperative refractions from MRSEs were calculated. The SRK/T, Haigis, BUII, Hill-RBF (version 3), and machine learning-based methods were evaluated.

### IOL power calculations

For the SRK/T and Haigis formulas, the constants were optimized using the training set. Optimization of the BUII calculation was not conducted since it has not been commonly available [12]. As the Hill-RBF version 3 which was optimized for SN60WF and MA60MA (Alcon) IOLs by utilizing pattern recognition by artificial intelligence together with a large global database [3], no optimization was applied.

**Fig. 2:**
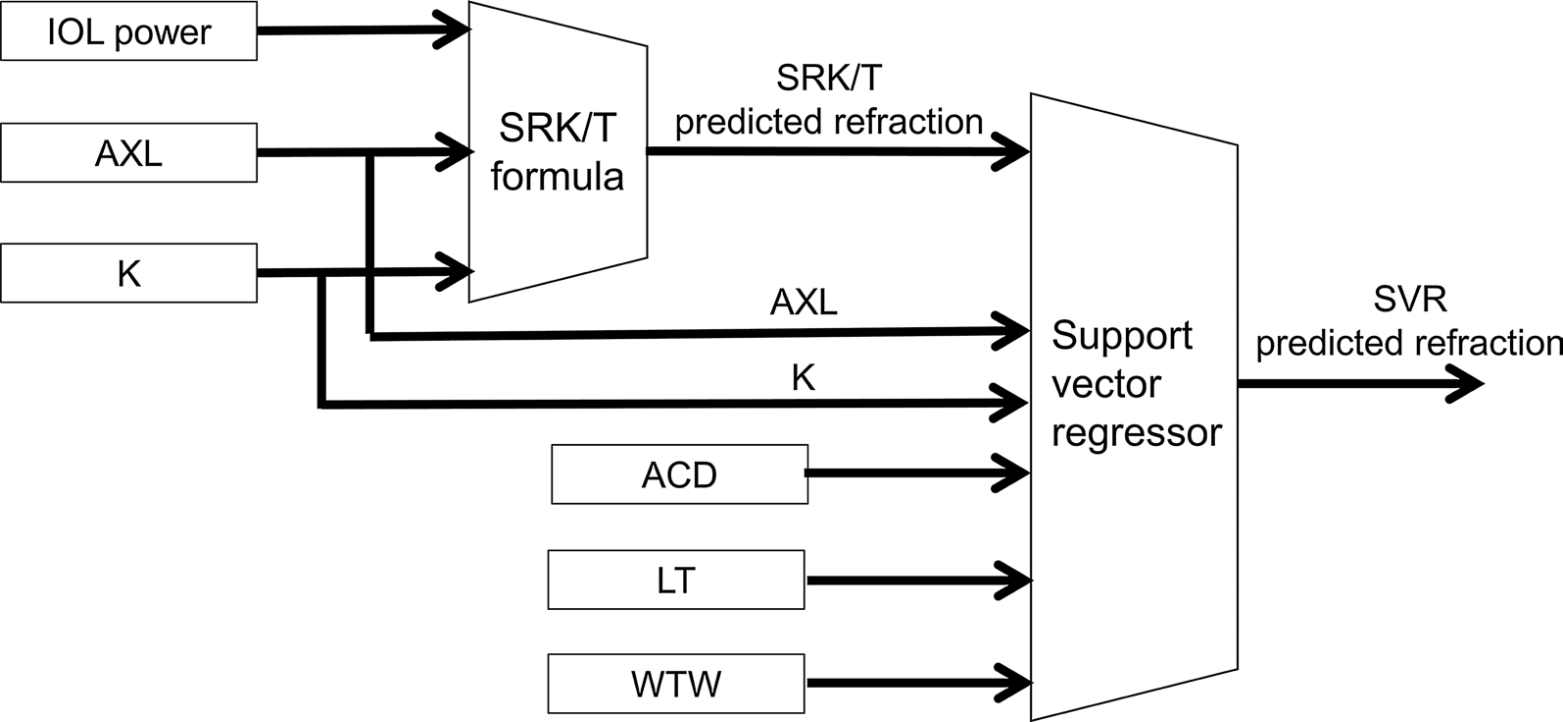
Architectural schematics of IOL power calculation using a support vector regressor (SVR). Inputs of SVR were predicted refraction results obtained with the SRK/T formula, axial length (AXL), mean keratometry (K), anterior chamber depth (ACD), lens thickness (LT), and white-to-white width (WTW)

The adapted calculator was designed using SVR [16]. Figure 2 shows the schematic architecture of an adapted calculator; AXL, K, ACD, LT, WTW and the predicted refraction obtained from the SRK/T formula were inputs. This calculator was designed for refining the outputs from the SRK/T with SVR machine learning with the training set. The calculator was created by utilizing the “scikit-learn” library (https://scikit-learn.org/stable/modules/svm.html#svm-regression) in the programming language Python 3. In training of SVR with RBF kernel, hyperparameters such as a C constant and shape parameter γ of the kernel function were tuned using a grid search for avoiding overfitting.

With the validation set, 5 kinds of calculations, i.e., the SRK/T, Haigis, BUII, Hill-RBF, and adapted methods were evaluated. To assess the accuracy achieved in practice situations, further optimization was not conducted. The means and standard deviations (SDs) of prediction errors were calculated. For absolute prediction errors, the median absolute error (MedAE) was obtained. The proportion of eyes within ± 0.25 D, ± 0.50 D, and ± 1.00 D prediction errors were calculated. In addition, the eyes of + 0.50 D or larger errors were also calculated as hyperopic errors are more severe with respect to a patient’s quality of life.

### Statistical analysis

Mean prediction errors were compared between the 5 calculations using repeated ANOVA followed by the Holm multiple comparisons, while the absolute prediction errors were examined using the Freedman test followed by Scheffé pairwise comparison. The percentages of eyes within ± 0.25 D, ± 0.50 D, and ± 1.00 D, and over + 0.50 D were examined using the Chi-squared test following the residual test. Associations of AXL, ACD, K, and WTW to prediction errors in the use of the adapted calculator were examined using linear regression analysis. *P* < 0.05 indicates a statistically significant difference.

## Results

The sample consisted of 1611 eyes from 1169 eligible patients. Among them, the validation set consisted of 400 eyes of 400 individual patients chosen at random, and the rest were used for the training set (1211 eyes of 769 patients). Demographic data for both sets are shown in Table 1. Although the K values were significantly different, the mean difference (0.22 D) was only 0.5% of the mean value and would be within the level of examination tolerance. There were no differences in other biometric parameters. For the powers of implanted IOLs and MRSE, no difference was found.

**Table 1:** Demographic data for the training and validation sets

Parameter	Training set mean ± SD (range)	Validation set mean ± SD (range)	*P* value*
N, eye/patient	1211/769	400/400	
Age (years)	72.8 ± 8.7 (24–92)	73.1 ± 9.8 (31–93)	0.65
Axial length (mm)	23.92 ± 1.54 (20.8–29.8)	23.92 ± 1.53 (21.1–32.0)	0.78
Anterior chamber depth (mm)	3.15 ± 0.40 (2.12–4.54)	3.15 ± 0.40 (2.15–4.06)	0.97
Mean keratometry (D)	44.35 ± 1.54 (37.3–49.7)	44.13 ± 1.63 (36.8–48.5)	0.02
Lens thickness (mm)	4.63 ± 0.44 (3.0–5.8)	4.63 ± 0.45 (3.3–5.9)	0.75
Central corneal thickness (μm)	518.4 ± 32.2 (365–634)	518.8 ± 33.6 (392–606)	0.84
White-to-white width (mm)	11.7 ± 0.4 (10.2–13.0)	11.7 ± 0.4 (10.2–12.9)	0.35
IOL power (D)	20.0 ± 3.9 (6.0–30.0)	20.2 ± 3.8 (6.0–28.0)	0.49
Postoperative MRSE (D)	− 0.39 ± 0.81 (− 4.88–1.25)	− 0.37 ± 0.86 (− 5.25–1.63)	0.72

*IOL* intraocular lens; *MRSE* manifest refraction spherical equivalent; *D* diopter

^*^ Unpaired t-test

With the training sets, the constants were optimized to be A = 119.18 for the SRK/T formula and a0 =  − 2.1245, a1 = 0.2032, and a2 = 0.2866 for the Haigis formula.

**Fig. 3:**
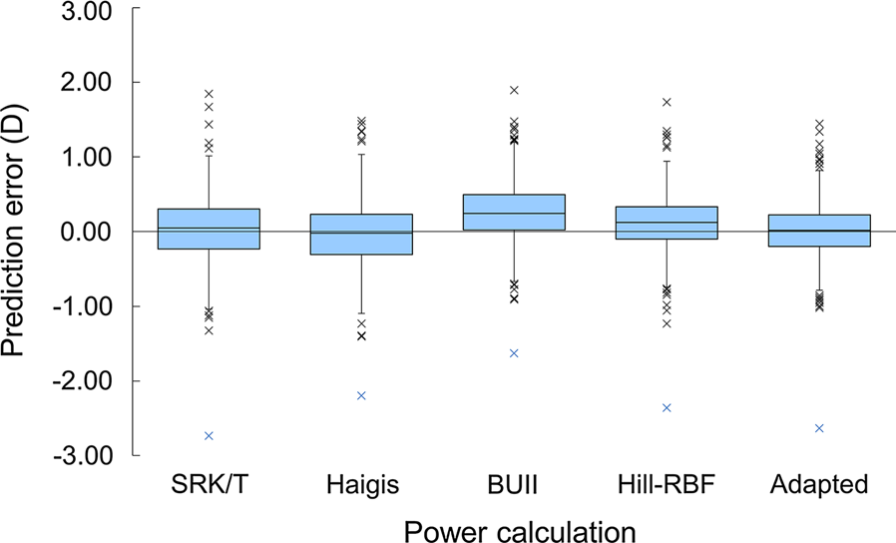
Box plots of prediction errors in the use of the 5 kinds of power calculations

In the validation set, the Hill-RBF was not available for 4 eyes due to being out of range. Figure 3 shows the distribution of the prediction errors from 396 eyes. There was one subject resulting in a myopic outlier (denoted as a “blue x” in Fig. 3), which corresponded to an eye with a short AXL (22 mm), a shallow ACD (2.43 mm) and an abnormality in corneal topography. Hence, this subject was also excluded from further analysis.

**Fig. 4:**
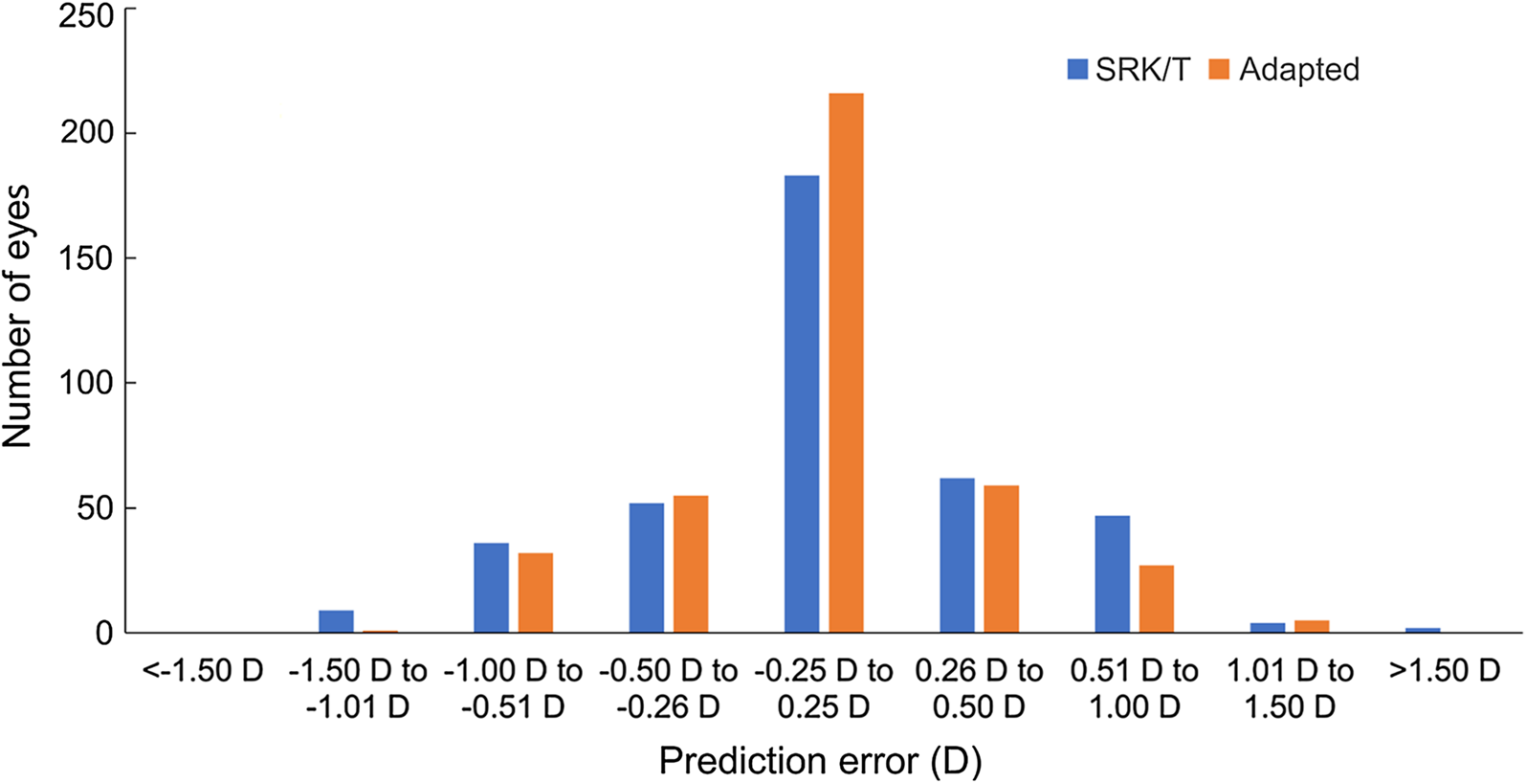
Distribution of prediction errors in the use of the SRK/T and adapted calculation

Table 2 lists the means and SDs of the prediction errors, the MedAEs, and the percentages within ± 0.25 D, ± 0.50 D, and ± 1.00 D, and over + 0.50 D. For the mean prediction errors, the SRK/T and adapted formulas resulted in lower mean values (*P* < 0.001, Holm multiple comparison) compared with others. Distribution of prediction errors in the use of the SRK/T and adapted formulas are shown in Fig. 4, which indicate that the adapted calculation reduced prediction errors in both the myopic and hyperopic directions. For the absolute prediction errors, the use of BUII resulted in higher errors than the other calculations (*P* < 0.010, Scheffé multiple comparison), and the adapted method showed lower errors than the use of the SRK/T, Haigis and BUII formulas (*P* < 0.011). No statistically significant difference was found between the Hill-RBF and adapted methods (*P* = 0.76).

**Table 2:** Means, SDs, MedAEs of prediction errors, and proportions of errors within ± 0.25 D, ± 0.50 D, ± 1.00 D, and greater than + 0.50 D in the use of 5 kinds of power calculations (N = 395 eyes)

Power calculation method	SRK/T	Haigis	BUII	Hill-RBF	Adapted
Mean (SD), D	0.02 (0.45)	− 0.03 (0.44)	0.25 (0.40)	0.12 (0.40)	0.01 (0.38)
MedAE, D	0.27	0.27	0.31	0.27	0.21
Within ± 0.25 D	46.1%	47.1%	41.0%^x^	46.6%	54.4%*
Within ± 0.50 D	75.2%	78.2%	70.9%^x^	79.5%	83.5%*
Within ± 1.00 D	96.2%	96.7%	96.2%	97.5%	98.5%
Greater than + 0.50 D	24.8%	21.8%	29.1%	20.5%	16.5%*

*BUII* Barrett Universal II; *SD* standard deviation; *D* diopter; *MedAE* median absolute error

Adapted denotes SRK/T formula adapted with support vector regressor

^*^ and ^x^: significantly larger or smaller than the mean levels, respectively (residual analysis)

**Fig. 5:**
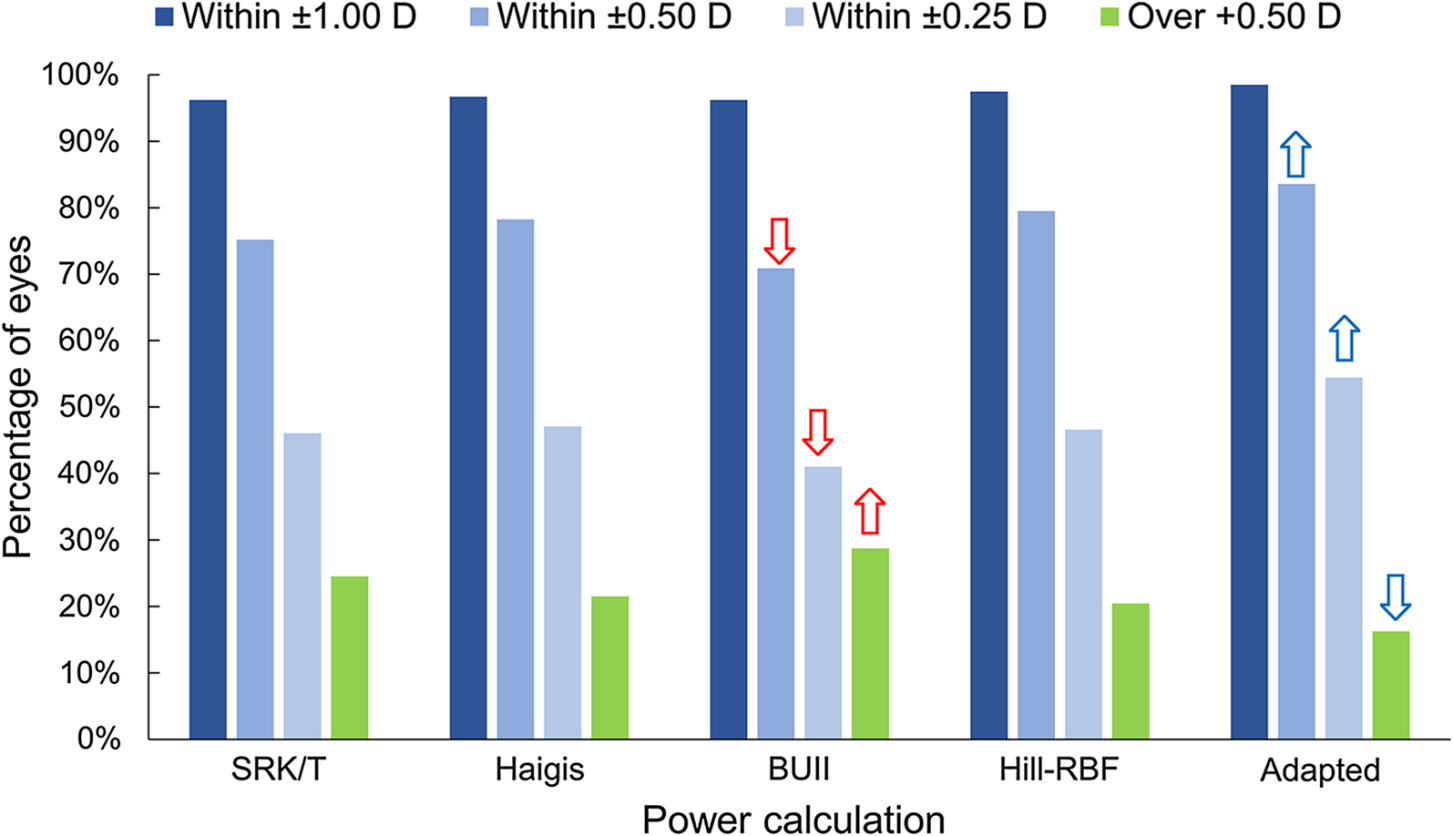
Percentages of eyes with errors within ± 0.25 D, ± 0.50 D, and ± 1.00 D as well as greater than + 0.50 D for the use of 5 kinds of power calculations (N = 395 eyes). Blue and red arrows denote significantly superior and inferior proportions of eyes compared with other calculations

The percentages of eyes within errors of ± 0.25 D, ± 0.50 D, and ± 1.00 D are shown in Table 2 and Fig. 5. No difference was found within ± 1.00 D errors. More eyes achieved errors within ± 0.50 D in the use of the adapted method (*P* = 0.0012, residual analysis), while the number of eyes within this error bound was significantly lower in the use of the BUII method (*P* < 0.001). The adapted method achieved more eyes with errors within ± 0.25 D than did others (*P* < 0.001). Hyperopic errors over + 0.50 D were significantly less with the use of the adapted method (*P* = 0.0012), but there were more eyes in the use of BUII (*P* < 0.001).

**Fig. 6:**
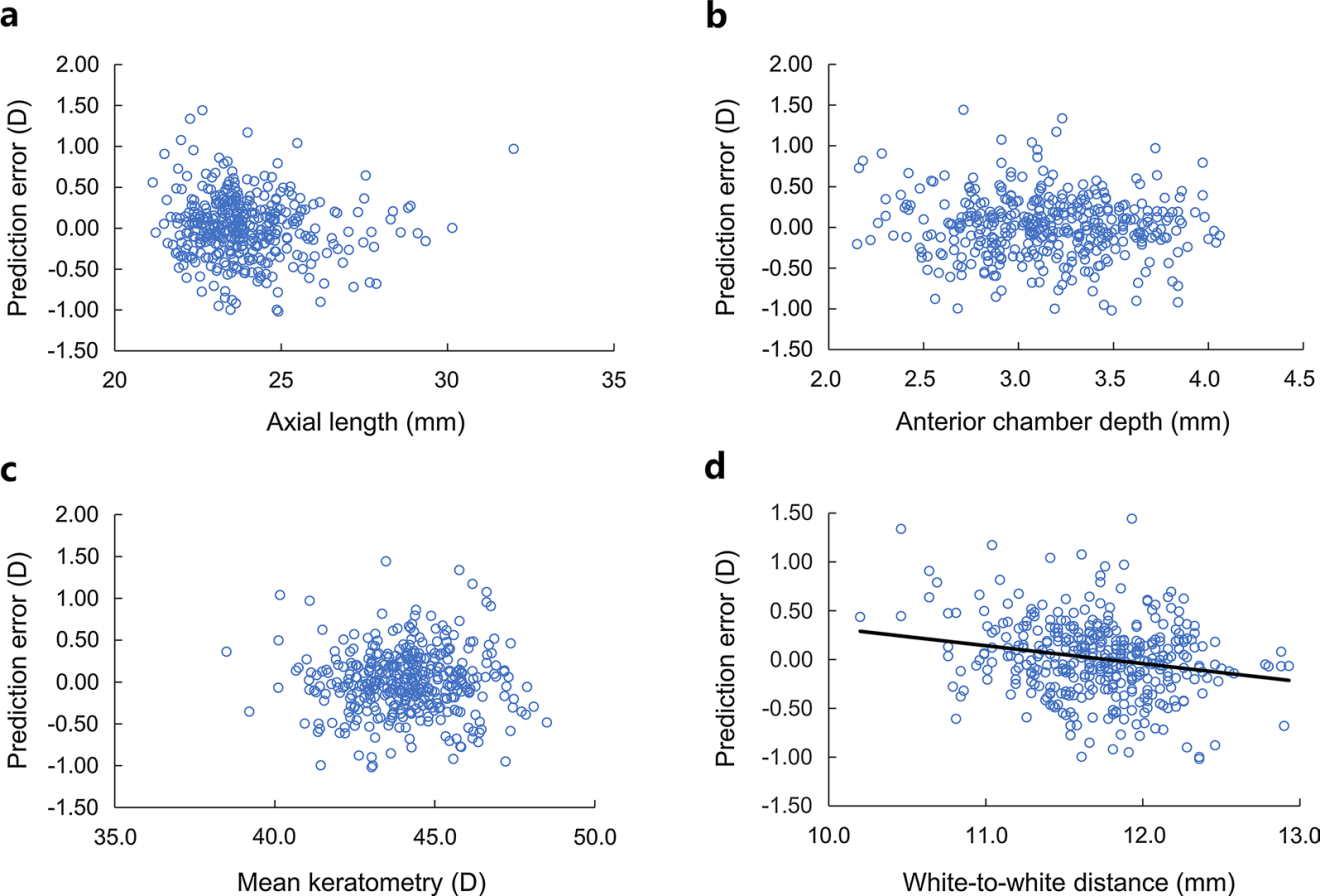
Relationship of axial length (**a**), anterior chamber depth (**b**), mean keratometry (**c**), and white-to-white (**d**) values to prediction errors in the use of the adapted calculation. There was no significant association except for white-to-white (*P* < 0.001, R^2^ = 0.044, linear regression analysis)

Figure 6 shows the relationship of AXL, ACD, K, and WTW to prediction error in the use of the adapter method. There was no significant correlation with AXL, ACD and K values. The prediction errors were correlated with WTW (*P* < 0.001, R^2^ = 0.044, linear regression analysis), and the resultant regression equation indicated no prediction error at WTW of 11.8 mm.

## Discussion

For this patient group, IOL power calculations adapted with the patient group data achieved better accuracy than the other formulas. For Japanese patients on Southern Kyushu Island, the performance of BUII was no better than that of the constantly optimized SRK/T and Haigis, supporting the importance of adaptation for the patient group. The accuracy improved with the use of SVR for refining the SRK/T with the training set, as presented in previous studies [17–20]. For Chinese myopic eyes, the machine learning-based calculation showed superior results to the BUII [20]. The findings indicated that adapting a power calculation formula for a patient group would be effective.

Machine learning and artificial intelligence have been applied for IOL power calculations. The theoretical optics-based Kane formula incorporates both a regression component and artificial intelligence using approximately 30,000 cases [5]. The parameters of the AL, keratometry, ACD, and sex are required. The Hill-RBF uses adaptive learning from large biometric data of eyes with SN60WF and MA60MA IOLs (Alcon), measured with an LS 900 optical biometer (Haag-Streit) [3]. Table 3 shows the performance of machine learning-based formulas. The performance in the use of the Hill-RBF version 2 and Kane formulas for Chinese eyes was lower than that for Caucasian eyes [17, 21]. Geometric differences in biometric parameters from the training dataset is one of the contributing factors. In the use of formulas developed with data from the patient group, such as XGBoost [6] and current SVR machine learning, the mean prediction errors, MedAEs, and percentages of eyes within ± 0.25, ± 0.50, and ± 1.00 D were comparable with the accuracy obtained with Caucasian eyes. The comparison of the current and previous performances indicated the influence of the patient group and the effectiveness of a patient group-based adaptation. In addition, it was anticipated that machine learning adaptation would be effective for the use of IOL differences from those used for training.

**Table 3:** Performance of machine learning-based formulas

Study	Carmona González D et al. (N = 260) [17]		Zhao J et al. (N = 53) [21]		Wei L et al. (N = 1450) [20]	Current
Calculation method	Hill-RBF*	SVR + MAR	Hill-RBF*	Kane	XGBoost	SRK/T + SVR
IOL	10 models	10 models	SBL-3	SBL-3	8 models	SN60WF
Country	Spain	Spain	China	China	China	Japan
Mean prediction error (SD), D	− 0.17 (0.40)	0.04 (0.30)	− 0.51 (0.61)	− 0.50 (0.60)	N.A.	0.01 (0.38)
MedAE, D	0.28	0.18	0.55	0.45	0.29	0.21
Within ± 0.25 D	48.1%	65.4%	24.5%	28.3%	43.9%	54.4%
Within ± 0.50 D	80.8%	90.4%	47.2%	52.8%	72.8%	83.5%
Within ± 1.00 D	100.0%	100.0%	81.1%	83.0%	99.1%	98.5%

*SVR* support vector regression; *MAR* multivariate adaptive regression spline; *XGBoost* extreme gradient boosting machine learning; *SD* standard deviation; *D* diopter; *MedAE* median absolute error; *N.A.* not available

* version 2

In the mean prediction errors with the adapted calculations, there was no association with the AXL, ACD, and K. While the AXL range was limited in the current study, the influence of AXL was minimal. In an evaluation of 828 patients in Spain, the mean prediction errors with the use of the Hill-RBF Version 2 and Kane formulas did not change relative to the AXL [22]. As the patient group was different from the one which produced previous results, the influence of AXL on the accuracy was not found in the adapted calculation. In contrast, there was a significant correlation with WTW, and the regression equation equaled to be zero at 11.8 mm. Compared with demographic of the patients, this result demonstrates that the adapted calculation successfully minimized WTW influence.

The adapted IOL power calculation was designed for refining the predicted refractions delivered from the conventional SRK/T formula, while most of the previous approaches did not utilize the conventional formulas [3, 5, 17, 19, 20]. As the training was focused on refining the prediction of the SRK/T formula, the accuracy was obtained with a limited size of the training dataset, as demonstrated previously [20]. The improvement in the use of SVR was higher than the use of constant optimization (Table 2), while increasing the inputs of biometric parameters (AXL, K, ACD, LT, and WTW) also contributed. It was anticipated that the current approach would be beneficial for adaptation for the patient group. Further evaluations are necessary for verifying it.

The effectiveness for other types of IOL was a concern. Previous assessments of machine-learning based calculations demonstrated the use for other IOL models with open-loop design [5, 17, 19]. In contrast, for IOLs with plate or loop haptics, postoperative position varies with the geometric difference in crystalline lens [23]. Small or shrinking equatorial diameter can displace the IOL posteriorly and induce hyperopic refractive errors. Unfortunately, such influences could not be adapted in the current approach since accurate lens geometry and quantitative evaluation of the influence are not available at this moment.

There were limitations in the retrospective evaluations. First, although most of biometric parameters were measured with a swept-source biometer, other geometric characteristics of the patient group, such as angle and diameter of crystalline lens, as well as the postoperative ACD and anterior capsulotomy, could not be examined in the retrospective design. Hence, the causes of larger errors in the use of BUII could not be evaluated. Next, most of the subjects were in the range of normal Japanese eyes: AXLs of 87.1% of eyes were between 22 and 26 mm in the validation set. In vergence-based formulas, the prediction errors increase in short and long eyes [6]. Further evaluations are required for short and long eyes. Moreover, it was not clear whether the SVR-based calculations would be effective for other IOL models. Conventional formulas allow the use of any IOL model by adjusting the lens-related constants. Similarly, the SVR-based method would accommodate other IOL models with the A-constant in the SRK/T calculation; however, it has not yet been examined. In addition, the accuracy for cases in which the biometry data are not within the range of the training set, are not predictable. In machine learning-based calculations, out of range data lower accuracy but increasing the size of the training set could circumvent this issue [3]. As the current approach used nonlinear regression [16] as well as data set restricted in the particular patient group, it is anticipated that such a risk and influence would be reduced. Lastly, it was of interest whether such a machine learning-based adaptation could be possible and practical. Currently, some popular machine learning techniques are available in free software environments, such as Python and R. In R, SVR was available in the package ‘e1071’ (https://cran.r-project.org/web/packages/e1071/index.html). Although it takes time to learn the software, adaptation for the patient group can very well be possible. However, there was no idea as to how much data from the patient group would be minimally required, owing to the nonlinear property for trainings.

## Conclusion

The current study demonstrated the effectiveness of the adaptation of IOL power calculation formulas utilizing the SVR and the patient group data. The adapted calculation would outperform the constant optimization and the use of latest formulas when there are geometric differences in the patients’ eyes.

## Data Availability

The datasets used and/or analyzed during the current study are available from the corresponding author upon reasonable request.
